# Cryopreservation and transplantation of ovarian tissue: results from one center in the USA

**DOI:** 10.1007/s10815-018-1315-1

**Published:** 2018-09-25

**Authors:** Sherman J. Silber, Michael DeRosa, Sierra Goldsmith, Yuting Fan, Leilani Castleman, Jeffrey Melnick

**Affiliations:** 1 Infertility Center of St. Louis, 224 South Woods Mill Road, Saint Louis, MO 63017 USA; 2 Whitehead Institute, Massachusetts Institute of Technology, Cambridge, MA USA; 3 Sun Yat-sen University of Medical Sciences, Guangzhou, Guangdong China; 4 St. Luke’s Hospital Pathology, 232 South Woods Mill Road, St. Louis, MO 63017 USA

**Keywords:** Ovary tissue, Fertility preservation, Ovary transplantation, Ovary tissue cryopreservation, Cancer and fertility

## Abstract

**Purpose:**

To report the results of cryopreserved ovary tissue transplantation for leukemia and other cancers, in a single US center.

**Methods:**

One hundred eight females between age 6 and (median age 24) 35 were referred for possible ovary tissue cryopreservation over a 20-year period, with either slow freeze or vitrification. Thus far 13 patients returned up to 18 years later to have their tissue transplanted back.

**Results:**

All 13 patients had return of ovarian function 5 months post transplant with regular menstrual cycling. AMH rose to very high levels as the FSH declined to normal. Four months later, the AMH again declined to very low levels. Nonetheless, the grafts remained functional for up to 5 years or longer. Ten of the 13 (77%) became spontaneously pregnant at least once, resulting in 13 healthy babies. A total of 24 healthy babies have been born 11 from fresh transplanted ovarian tissue and 13 from cryopreserved transplanted ovarian tissue.

**Conclusions:**

(1) Ovary tissue cryopreservation is a robust method for preserving a woman’s fertility. (2) Cortical tissue pressure may be a key regulator of primordial follicle arrest, recruitment, and ovarian longevity. (3) This is the only such series yet reported in the USA.

## Introduction

A series of 108 cases of ovary tissue cryopreservation initiated in 1997 gave us an opportunity to assess its efficiency for young women about to undergo sterilizing cancer treatment [1, 2]. Successful fresh and cryopreserved ovarian cortex transplants in humans were first published in 2004 and 2005, as case reports, and many other case reports have subsequently followed [3–22]. The first human applications were preceded by a long history of animal experimentation. As far back as 1954, Deanesly showed in rats, and in 1960, Parrott showed in mice, that ovarian tissue could be successfully frozen and autografted resulting in live births [23, 24]. Candy et al. showed these mice had a normal reproductive lifespan [25]. Interest in human applications began after Gosden’s’ report of successful pregnancies in sheep in 1994 [26]. Interest in cryopreserved ovarian cortical transplantation is rapidly growing, but systematic reports have been published from only a few centers [8]. Despite this great interest, there is a paucity of consistent series (none from the USA) reported from one center in which the expectation of success rate for this procedure can be gleaned [27–29].

The primary impetus for this procedure has been to cryopreserve ovarian tissue before sterilizing cancer treatment, with the objective of transplanting the tissue back after cancer has been cured, thus allowing patients to preserve their fertility. It is also possible that grafts taken from young women with cancer could be used in the future to delay their menopause [22, 30–33]. This latter possibility of preserving not only fertility, but even hormonal function against the natural decline caused by aging has even been speculated as a possible indication for young healthy women as well [34–37]. Most published research in this field consists of case reports of cryopreserved transplants only, because oncologists refer very few cases, and there has been a fear of re-introducing cancer cells, which has only recently been dispelled [27–29, 38]. Thus far there have been no cases reported of transmission of cancer either in our series or elsewhere in the world. A worldwide survey of 37 babies born from cryopreserved transplants still could not establish a clear success rate [22]. Here, we report a single series (though small) of cryopreserved transplants from one center, carried out with the same technique and assessed uniformly over follow-up. This is the only series we are aware of to be reported from the USA.

## Materials and methods

### Patients

Over a period from 1997 to 2017 (20 years), 108 females between age 6 and 35 years were referred for possible ovary tissue freezing for fertility preservation. Ninety-two (85%) of these women underwent unilateral oophorectomy and cryopreservation either by slow freeze or vitrification. Sixty-six were for cancer, 5 for threatened premature ovarian failure (ovary tissue of discordant identical twin that was cryopreserved for her sister), 9 for social reasons, and 12 for a variety of conditions including Turner’s syndrome, multiple sclerosis, endometriosis, aplastic anemia, a daughter born with no ovary, or massive bilateral ovarian teratoma. Hodgkin’s disease accounted for 20 of the cancer cases (30%), breast cancer 13 (20%), leukemia 7 (11%), and non-Hodgkin’s lymphoma 5 (8%). The rest of the cancer cases (21) were a wide variety of less common cancers such as Ewing’s sarcoma, embryonal sarcoma of liver, colon cancer, calf sarcoma, spinal cord tumor, dysgerminoma, medulloblastoma, rhabdomyosarcoma, stomach cancer, carcinoid tumor, and brain cancer. Social reasons included not being ready to have children and wishing to have more children perhaps at a later date. Those who chose ovary freezing for social reasons did so before oocyte freezing was widely accepted, or because they were just too occupied in their hectic life to find time for three cycles of ovarian stimulation and oocyte retrieval, and did not want to go through hormonal stimulation. Nineteen of the cancer patients underwent slow freeze prior to September 2007, and 47 subsequent cases underwent vitrification of their ovarian tissue. Six of the 66 (10%) cancer patients have died, and 54 either underwent transplantation, or are prepared eventually to undergo transplantation. All patients were counseled in detail with the advice that the transplant might not ever be performed, or might not function. All underwent IRB consent.

Thirteen of the 92 ovary freeze cases (14%) have come back to have their ovary tissue thawed and transplanted back. Of those 13, the four most recent cases had been cryopreserved by vitrification, and the other 9 had been frozen by slow freeze. Ten were cancer survivors, and three were POF patients who had frozen ovary tissue from an identical twin sister. The 9 slow freeze cases had their ovary tissue frozen before 2007, and so naturally they composed the majority (9) of the cases of cryopreserved tissue thaw and that were transplanted. The other four cases of frozen ovary transplant had their tissue cryopreserved after 2007, and so these were vitrification cases. In addition to these cryopreserved ovary tissue transplant cases reported here, there have been 11 fresh transplants either between identical twins or allografts, that have already been reported, for a total of a large series of 24 ovary transplants that have been performed at one center with one technique [1]. All patients were menopausal for 3 to 20 years prior to the transplant. Three of the nine women undergoing transplant of their frozen tissue had leukemia, but their tissue was cryopreserved when they were in remission prior to their bone marrow transplant [38]. Assessment of multiple fragments by histology and immunochemistry by oncology and pathology departments revealed no tumor cells.

### Cryopreservation

The technique for slow freeze and thaw has not changed since the original description by Gosden et al. in 1994 [18, 26]. Slow freezing is the earliest approach to ovarian cryopreservation. The cortex is removed from the medulla, divided into multiple strips of 1 cm by 2 cm by 0.1 cm and transferred to cryovials after incubation in 1.5 mol/L 1,2-propanedial ethylene glycol or DMSO, and 0.1 mol/L sucrose with 10% SSS at 37 °C for 30 min; then, 0.2 mol/L sucrose for 5 min, and after that cooled by computerized lowering of temperature [39]. Cooling was first at 2 °C/min to − 9 °C and then seeded. Thereafter, cooling rate was − 0.3 °C/min down to − 40 °C. Then, − 10 °C/min to − 140 °C and then plunged in liquid nitrogen. Thawing is performed rapidly (100 °C/min) in a warm bath and tissue is trimmed under an operating microscope before transplantation [6, 18, 26, 39]. This slow freeze method has worked quite well, and most of the many pregnancies and live births achieved so far have been with slow freeze. However, since 2007, we have used vitrification, because in vitro viability analysis studies shows no oocyte loss with vitrification, and a 50% oocyte loss with slow freeze [9, 10, 40]. Vitrification may show no difference in percent of morphological normal primordial follicles, but does result in less DNA damage and should cause and therefore probably less decrease in viability [41].

Nonetheless, slow freeze has had equally robust for ovarian tissue cryopreservation. One possible speculation is that if you lose half of 100,000 or 200,000 eggs, it might not affect the clinical result, because of a conceivably lower rate of primordial follicle recruitment. Whatever the reason, we cannot claim better clinical results with vitrification.

The method of both cooling and warming for vitrification are as follows [9, 10, 40]. For vitrification, the cortex tissue of each ovary is cut into slices 10 mm by 10 mm by 1 to 1.5 mm. The tissue slices are initially equilibrated in 7.5% ethylene glycol (EG) and 7.5% dimethyl sulfoxide (DMSO) in HEPES-buffered handling media supplemented with 20% synthetic serum substitute (SSM) for 25 min. Then, a second equilibration is performed in 20% EG and 20% DMSO with 0.5 M sucrose for 15 min or until the slices descend to the bottom of a 50-ml centrifuge tube indicating complete absorption of the cryoprotectants. The tissues are then placed on a thin metal strip (Kitayzato Bio Pharma, Japan) which is then plunged directly into sterile liquid nitrogen and inserted into a “closed” tube which contains liquid nitrogen for storage.

For thaw, the metal strip is immersed swiftly into 40 ml of 37 °C HEPES-buffered handling media supplemented with 1.0 mol sucrose for 1 min, and then 40 ml of 0.5 mol sucrose for 5 min at room temperature and washed twice for 10 min in standard handling media. Standard H+E histology and dye exclusion is performed to check for viability and presence of cancer cells.

### Transplant surgical technique

**Fig. 1:**
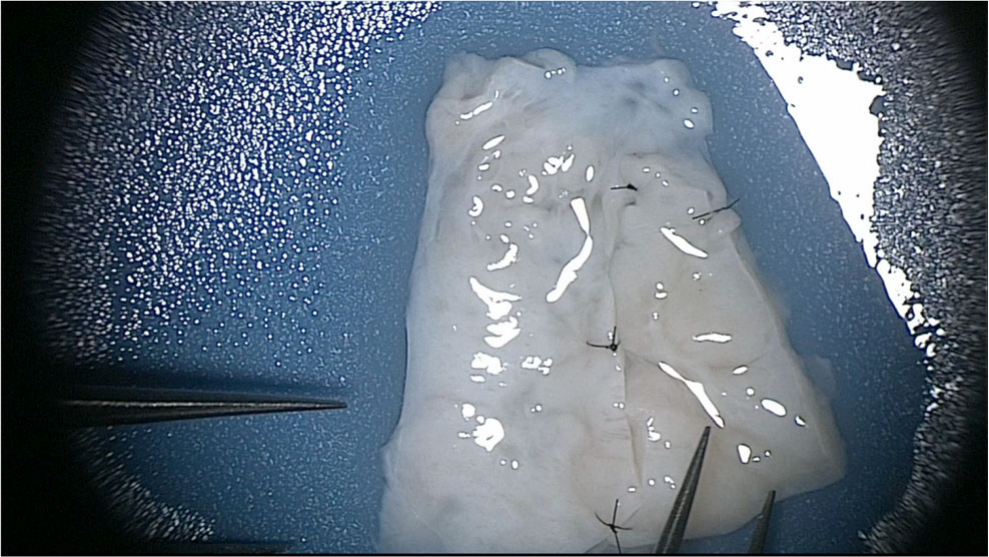
Ovary transplant tissue quilting

The transplant technique has not changed since our first fresh transplant in 2004, which has been well described [18]. The ovary cortical strip after thawing is treated as though it were a full thickness skin graft (1–1.5 mm in thickness, and 1 cm by 1 cm size pieces). On average, only 25% of stored tissue was thawed and transplanted. The thawed strips or slices are then quilted together with a 9-0 nylon sutures and applied to the denuded ovarian medulla of the remaining intact ovary, (Fig. 1). Micro-hematoma formation under the graft as well as adhesions were avoided by meticulous micro-bipolar cautery with pulsatile heparinized saline irrigation, and multiple micropressure interrupted stitches of 9-0 nylon. Constant pulsatile irrigation is used to prevent adhesions, so as to allow spontaneous pregnancy with no need for IVF. All transplants were therefore orthotopic, with a healthy fallopian tube in good position for ovum pickup.

**Fig. 2:**
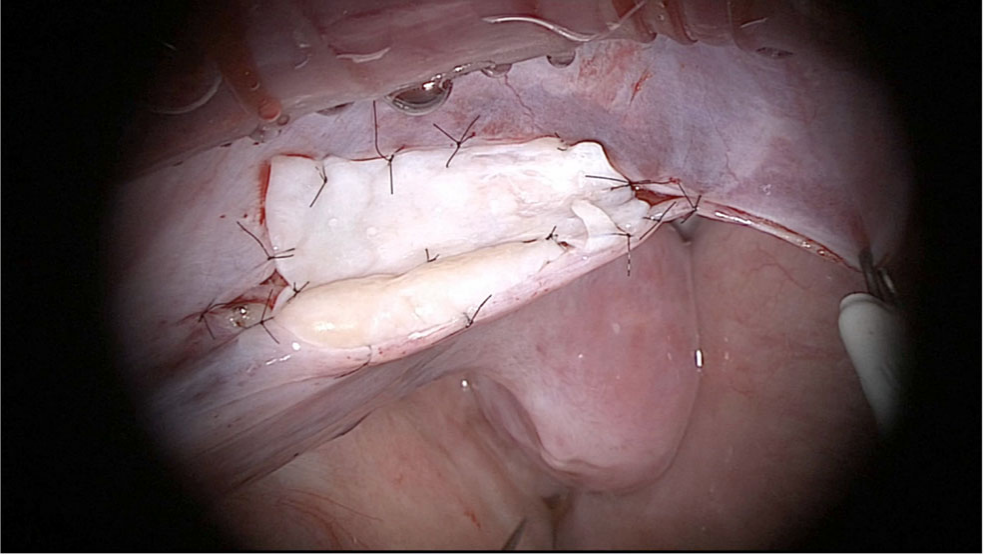
Ovary *transplant*

Before transplanting to the denuded ovarian medulla, the slices of thawed ovarian tissue were quilted together into one graft using 9-0 nylon interrupted sutures, before the patient was taken to the operating room and anesthetized (Figs. 1 and 2). All procedures were performed with minilaparotomy and an operating microscope. The reasons for using minilaparotomy over laparoscopy or robot are as follows: (1) we used an operating microscope with 9-0 nylon interrupted sutures and continuous irrigation with heparinized saline to avoid adhesions which could interfere with spontaneous pregnancy, and (2) we inject the operative site with a combination of ropivacaine toradol, and morphine, which provides 3 days of local anesthesia and minimal pain. So it is a quick outpatient procedure despite not being laparoscopic.

### Postoperative follow-up

All patients underwent monthly hormone evaluation (FSH, estradiol, and AMH) and recording of the return of menstrual cycling. Once menstrual cycling returned, hormonal measurements were made on day 3 of each cycle. All patients were allowed to conceive spontaneously, and treatment intervention such as IVF or hormonal stimulation was not employed. All cases were approved by the IRB of St. Luke’s Hospital in St. Louis Missouri. This is a prospective series but not a randomized control (RCT) study.

## Results

All 13 cases had return of ovarian function from 4 to 5 months after transplantation, as determined by return of FSH to normal levels, and regular menstrual cycling, similar to what was previously reported with fresh ovary tissue transplantation. At the same time that FSH returned to normal or near normal levels, the AMH rose to high levels, and then fell to very low levels after 4 more months.

Eight of the 13 grafts were still functioning from 28 to 62 months after surgery. The other 5 grafts ceased functioning from 22 to 51 months. The longest functioning graft from slow freeze was 56 months and is still functioning. The longest functioning graft from vitrification was 62 months and is still functioning. The oldest female at the age of freeze was 31 years, and the oldest at the age of transplant was 39 years. No correlation was possible between age and duration of function, or live baby rate. But of course all recipients were between 19 and 31 years of age at the time of freeze for a median age of 24 (Tables 1, 2, and 3).

**Table 1:** Overall results and age

Date of transplant	Age at transplantation	Age at freeze	Diagnosis	Pregnant	Live birth or ongoing	Time until pregnancy (days)	Miscarriages	Duration of ovarian function (months)
3/6/07	26	24	POF	Yes	Female	174		23 (ended)
1/13/09	31	20	Hodgkins	Yes	Male	272		29 (ended)
6/9/09	29	24	POF	Yes		276	1	19 (ended)
6/17/11	33	20	Hodgkins	No				38 (ended)
10/12/12	33	31	MS	Yes	Female	481		67 (ended)
3/29/13	32	25	POE	Yes	Female	243		26 (ended)
4/5/13	33	30	Brain cancer	Yes	Male	665		61 (still functioning)
4/12/13	25	18	Leukemia	Yes	Male	502		61 (still functioning)
				Yes	Female	998		
				Yes	Female	1578		
10/1/13	29	28	Synovial sarcoma	No				56 (still functioning)
10/7/13	39	24	Leukemia	Yes	Female	1287		56 (still functioning)
7/21/15	28	25	Leukemia	No				34 (still functioning)
8/5/15	32	21	Hodgkins	Yes	Female	343		33 (still functioning)
9/18/14	36	20	Hodgkins	Yes	Female	473		44 (still functioning)
				Yes	Female	908		
					Female			
Totals	13 cases	13 babies	10 pregnant (77%)		1 miscarriage		10 females 3 males	4 vitrification 9 slow freeze

**Table 2:** Overall results vitrified versus slow freeze

Pregnancy after frozen autografts									
Vitrified slow freeze	Duration of ovarian function (months)	Date of transplant	Date of first menstruation post OT	Diagnosis	Miscarriage	Baby born	Ongoing	Girl	Boy
Slow freeze	23 (ended)	3/6/07	9/19/08	POF		1		1	
Slow freeze	29 (ended)	1/13/09	6/7/09	Hodgkins		1			1
Slow freeze	19 (ended)	6/9/09	11/28/09	POF	1	0			
Slow freeze	38 (ended)	6/17/11	11/15/11	Hodgkins		0			
Vitrified	67 (ended)	10/12/12	3/2/13	MS		1		1	
Slow freeze	26 (ended)	3/29/13	4/5/13	POF		1		1	
Vitrified	61 (still functioning)	4/5/13	12/27/13	Brain cancer		1			1
Slow freeze	61 (still functioning)	4/12/13	1/1/14	Leukemia		3		2	1
Vitrified	56 (still functioning)	10/1/13	12/19/13	Synovial sarcoma		0			
Slow freeze	56 (still functioning)	10/7/13	3/6/14	Leukemia		1		1	
Vitrified	34 (still functioning)	7/21/15	11/15/15	Leukemia		0			
Slow freeze	33 (still functioning)	8/5/15	10/28/15	Hodgkins		1		1	
Slow freeze	44 (still functioning)	9/18/14	2/2/15	Hodgkins		3		3	
Totals	13 cases	13 babies	10 pregnant (77%)	1 miscarriage	10 females 3 males		4 vitrification 9 slow freeze

**Table 3:** Leukemia cases

Ovary issue freeze transplants leukemia								
Date of transplant	Age at transplantation	Age at freeze	Diagnosis	Pregnant	Live birth	Time until pregnancy (days)	Miscarriages	Duration of ovarian function (months)
4/12/13	25	18	Myeloproliferative (blood disorder)	Yes	Yes	502		56 (still functioning)
				Yes	Yes	998		
				Yes	Yes	1578		
10/7/13	39	24	Acute lymphocytic leukemia	Yes	Yes	1287		50 (still functioning)
7/21/15	28	25	Acute myeloid leukemia	No				29 (still functioning)
Totals	3 cases	4 babies	4 pregnancies 2 became pregnant (67%)	0 miscarriage			Average age (30 years old)	

Nine of the 13 transplants resulted in spontaneous pregnancy and delivery of at least one live healthy baby (69%). In one case, three singletons thus far have resulted from one transplant, and in another case, two singletons and two spontaneous twins have resulted (no patients underwent IVF). There have thus been a total of 13 live, healthy babies from spontaneous pregnancy in these 13 cases. There has only been one miscarriage (10%) (Tables 1, 2, and 3).

Eleven babies have resulted from nine cases of slow freeze ovarian tissue, and two from four cases of vitrified ovarian tissue. Thus, 9 of the 13 cases resulted in at least one live birth spontaneous pregnancy (69%), one with 3 babies and one with four babies thus far. All of the four vitrified tissue cases are still functioning and four of the nine slow freeze cases are still functioning, which just means that the earliest cases were all slow freeze. All of the offspring are normal and healthy.

Three of the transplants were leukemia cases, for which their oncologist gave approval. Two of the three resulted in spontaneous pregnancy and delivery of four healthy babies (Tables 1, 2, and 3). There has been no recurrence of leukemia, and in fact no recurrence in any of the cancer cases. These were among the first cases of success for leukemia patients having babies from transplanting their frozen tissue although the first such case (from Israel) was published in 2017 (28). The first leukemia case in this series was frozen in 1997 and transplanted back in October 2013. Her baby was born in November 2017. The second leukemia case was frozen in 2006 and transplanted back April 2013, and her first of three babies was born in May 2015.

## Discussion

Young women or even prepubertal girls with cancer are likely to lose all ovarian function and be sterilized by their cancer treatment, which in the current era affords a high cure rate [30–33]. Since the report by Gosden et al. in sheep with frozen ovary tissue transplantation and the first reported cases in 2005 in humans there has been intense interest in preserving the fertility of these cancer survivors [6, 11, 18, 26]. Our first ovary freeze for cancer patients was in 1997. We did not begin transplanting back thawed ovarian tissue until 10 years later in 2007. It normally requires a long time before these women wish to have their cryopreserved ovary tissue transplanted back, as women in the modern era often delay marriage and childbearing, and they also want to wait until they have some assurance their cancer is cured [42]. So it requires many years of follow-up of frozen ovary tissue cases before results become available, and few centers have accumulated much experience therefore. Throughout the world, despite the strong interest and the desirability of a young woman preserving her fertility, there have not been many large series of cases from a single center, and none from the USA.

There have now been over 100 babies born around the world from ovary tissue transplantation in cancer survivors, with no reports of transmission of cancer except possibly from an ovarian cancer [27–29, 43]. So there is less fear now of transplanting this tissue back. Thus, one would expect cases like these to increase in number. Nonetheless, there are no such series from any one center in the USA. With the reluctance of oncologists, and the paucity of expertise, ovarian tissue cryopreservation is still deemed as “experimental” by ASRM, and insurance does not pay [44]. All of our patients were therefore taken on at no charge. Our current report of relatively robust results in a small but well-studied series might generate more enthusiasm in the USA to help these patients.

The unique features of this report include (1) the robustness of results with this technique, (2) the inclusion of both slow freeze and vitrification, (3) among the first successful results with three leukemia patients delivering four healthy babies, (4) most importantly, a well-studied series from one center (and the only such center) in the USA. The advantages of ovary cryopreservation over oocyte vitrification for cancer patients include no delay of cancer treatment, the avoidance of ovarian stimulation, and resumption of endocrine function.

None of our cases who were cured of cancer and came back to have ovary tissue transplanted back had any tumor cells in samples of their thawed ovary cortex [8, 29, 45]. The remarkable absence of ovarian metastasis might speculatively be due to the fibrous, avascular nature of the ovarian cortex [38]. The ovarian cortex of the ovary is identical to the tunica albuginea of the testis, and leukemia cells in prepubertal boys are rarely found in the tunica albuginea of the testis even though seminiferous tubules usually are filled with leukemia [38, 46, 47]. The reason why fetal ovarian follicles invade the fibrous cortex and become primordial follicles is that the dense fibrous tissue of the cortex is needed to suppress resting follicles from developing all at once prematurely [1, 48–51]. Without the ovarian cortex, initiating the formation of primordial follicles, fetal oocytes would continue in meiosis and be completely depleted by birth [49–52]. The dense fibrous tissue of the ovarian cortex not only controls follicle development but also represents a relatively inhospitable location for cancer cells. So prepubertal boys with leukemia usually have metastasis to the testis, but not to the tunica albuginea of the testis [38, 46, 47]. Furthermore, after initial chemotherapy for leukemia, when the patient is in temporary remission, there are no viable leukemia cells at all in her ovarian cortex [38]. Our three leukemia patients in this series had their ovary removed and cryopreserved while they were in remission before their bone marrow transplant. There has been no recurrence in these or any of our transplants. Meirow reports a similar experience [27, 28].

**Fig. 3:**
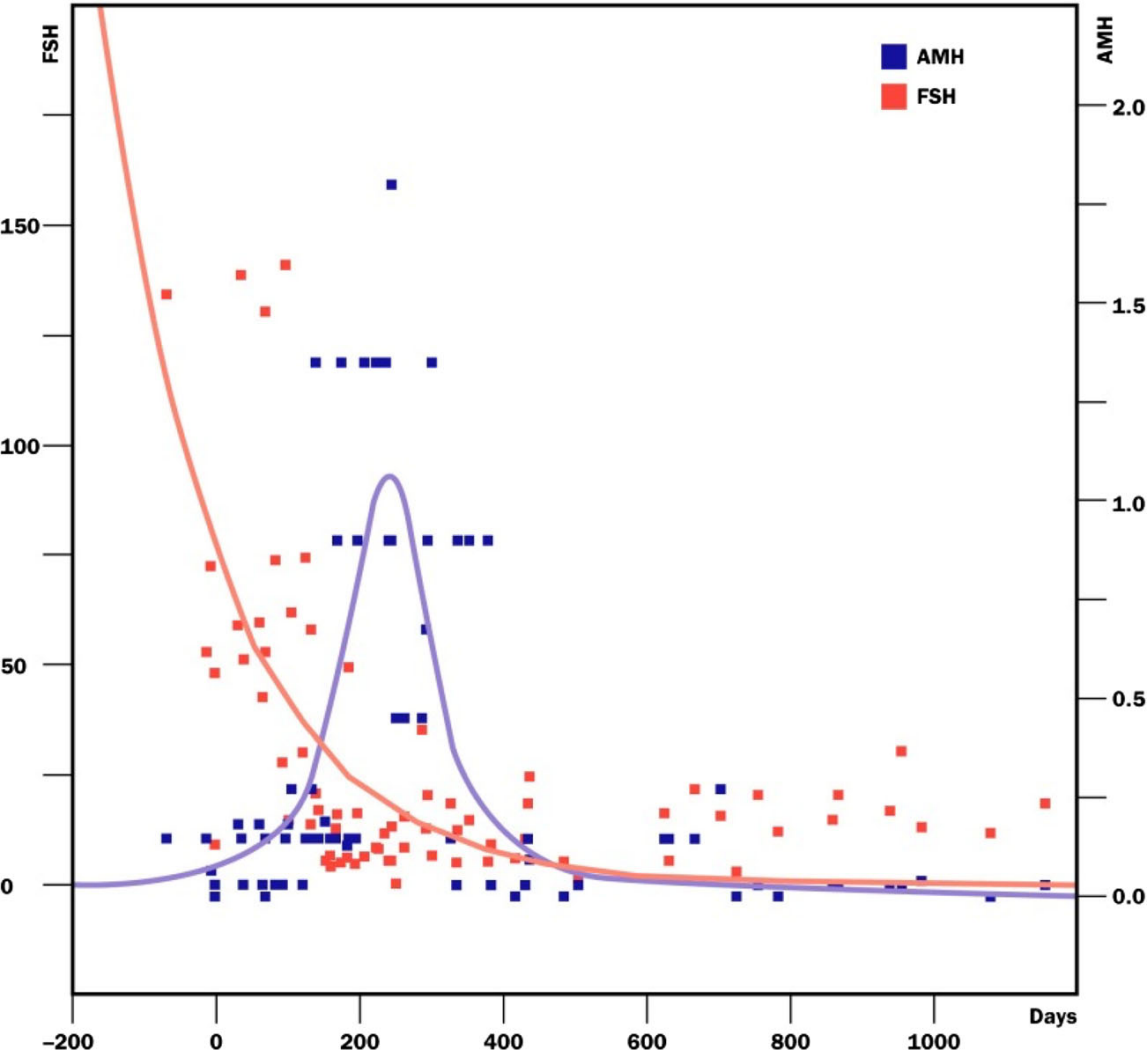
Composite dot graph summarizing the return of FSH (mIU/ml) to normal and the rise of AMH (ng/ml) and subsequent decline after frozen transplant

The return of FSH to near normal at 4 or 5 months indicates that this is the period of time required for primordial follicles, once recruited, to develop inwardly toward softer tissue, into the antral and ovulatory stage (Fig. 3). The concomitant rise of AMH followed by a drop to very low levels suggests a massive over-recruitment of follicles and subsequent depletion [54]. An alternative theory one could propose is that the high FSH at the time of transplant could have caused an over-recruitment of antral follicles as in ovarian hyperstimulation. However, this theory would not be consistent with a huge loss of ovarian reserve subsequently. Furthermore, a high FSH will not produce a higher AMH if the ovarian reserve is low. Only an over-recruitment of primordial follicles months earlier can do that.

Giving huge doses of gonadotropin to patients with low ovarian reserve will not increase the yield of eggs. However, these transplanted slices of ovarian cortex continue to function normally for many years despite a low AMH, as demonstrated in this series, because of a decreased rate of primordial follicle recruitment that occurs when there is decreased ovarian reserve [55–59].

After orthotopic frozen ovarian cortex auto-transplantation, all patients simply were able to attempt to conceive naturally without any other intervention. Because the ovary cortex was cryopreserved at a young age, there was no need to rush into IVF. Some did not conceive until 3 or 4 years after the transplant, possibly due to the effects of radiation or chemotherapy on uterine receptivity. But most did conceive successfully, and it is not clear that rushing into ancillary treatment or IVF would have helped the natural process, since the ovarian reserve was very low. But even with this very low AMH and low ovarian reserve, the cortical slices functioned for many years, and the majority of slices still remain frozen and ready for the future, when the current ovarian reserve disappears.

In a larger scientific context, this clinical experience reveals data on primordial follicle recruitment, and the convoluted difference between male and female germ cell development. The ovarian cortex participates in the locking and the unlocking of the primordial follicle, and until the transplant is healed, we suspect this underlying mechanism to be disrupted [49–51]. Intrinsic tissue pressure may be one mechanism at work to control primordial follicle status [53, 54, 60].

Primordial follicle arrest is the key to saving the oocyte from disappearing after the fetal initiation of meiosis and the continuation all the way through meiosis with subsequent apoptosis [49–52]. It is also the key to the cautious gradual release every month of oocytes in the adult to develop over 4 months into gonadotropin-sensitive antral and graafian follicles, sparing the resting oocytes from sudden total depletion [53]. After this depletion of resting follicles is halted, the ovarian transplant then proceeds to function surprisingly for many years quite well despite a very low AMH and a low remaining number of follicles. That is because as the ovarian reserve goes down, the rate of primordial follicle recruitment in a compensatory way also goes down. Huge population studies have indicated that unilateral oophorectomy does not cause much of an earlier menopause [55]. However, this assumption has been contradicted by a recent IVF study [56]. Nonetheless, this recent IVF study does not affect the finding of reduced primordial follicle recruitment in the face of reduced ovarian reserve. The less the number of remaining oocytes, the better the primordial follicles are able to maintain their locking mechanism and, as a result, limit the number of resting follicles allowed to activate and hence maintain follicle reserve [1, 48–51, 61–65].

The impression this series gives is the robustness of this “experimental” procedure. Our high success rates are most likely aided by having tissue only from younger women with no prior history of infertility. This is a relatively small series compared to the impressive experience of the Belgian, Israeli, Spanish, and Danish centers. Nonetheless, our live baby rate for these otherwise sterile cancer survivors and the obvious effectiveness of standard slow freeze (despite a previously demonstrated high oocyte loss compared to vitrification) for ovarian tissue cryopreservation, testifies to its robustness and simplicity.
